# Melatonin receptor 1 signaling modulates the metabolic response to high-fat diet in female melatonin-proficient mice

**DOI:** 10.3389/fendo.2026.1884802

**Published:** 2026-09-08

**Authors:** Varunika Goyal, Gianluca Tosini

**Affiliations:** Department of Pharmacology and NSF Center for Circadian Rhythmicity and Sleep Homeostasis, Morehouse School of Medicine, Atlanta, GA, United States

**Keywords:** dyslipidemia, female, high fat diet, insulin, melatonin, melatonin receptors

## Abstract

Melatonin plays an important role in the regulation of several physiological functions, including the regulation of circadian rhythms, sleep and reproduction. Recent studies have also reported melatonin signaling via its G-protein coupled receptors (namely MT1 and MT2), affects glucose metabolism, insulin sensitivity, and blood lipid levels. However, most of these studies have been conducted with melatonin-deficient male mice. In the present study, we investigated the effects of a low-fat diet (LFD) and high-fat diet (HFD) on liver biology and transcriptome using melatonin-proficient female mice (C3H-f+/+) and MT1 knockout mice. Our results demonstrate that MT1 deletion alone does not cause notable changes in weight, lipid profile or hepatic lipid accumulation, whereas the combination of MT1 deficiency and HFD exposure results in significant changes in glucose tolerance, serum lipid profiles and lipid accumulation in the liver. In addition, removal of MT1 signaling also induced significant changes in the liver transcriptome that were exacerbated by exposure to HFD. Finally, our data indicate that MT1 deletion was associated with changes in estrogen-related hepatic transcriptional pathways and circulating estradiol levels. These results suggest that MT1 signaling contributes to the metabolic and hepatic response to HFD in female mice. Our study also suggests that these effects may be associated with changes in estrogen-related signaling observed in MT1 KO mice under HFD conditions, although further studies are required to determine causality.

## Introduction

Melatonin is synthetized at night and is responsible for the regulation of circadian rhythms, sleep and reproduction in vertebrates (1–3). Several recent investigations have also demonstrated the involvement of this hormone in the regulation of metabolism (4, 5), blood glucose levels and metabolism (6–8), insulin sensitivity (6, 9, 10), and lipid homeostasis (11, 12). These effects are mediated via G protein-coupled melatonin receptors, such as Melatonin Receptor 1 (MT_1_) and Melatonin Receptor 2 (MT_2_), both of which are widely expressed in tissues throughout the body (13) and in the brain (14).

Previous work from our laboratory using melatonin proficient mice (C3H-f+/+) has reported an important role of melatonin signaling - mostly via MT1 - in the regulation of insulin sensitivity, glucose homeostasis and lipid processing. Indeed, these studies have shown that removal of MT1 leads to an increase in glucose tolerance and insulin resistance (15, 16), leptin regulation and energy balance (17, 18) and disruption in lipid metabolism (19, 20).

However, except for one study (10), all other studies have been performed in male mice and little attention has been placed on the possible role that melatonin signaling may play in female mice.

While metabolic pathways are fundamental across sexes, emerging evidence suggests significant sexual dimorphism in metabolic regulation. These studies have highlighted distinct metabolic profiles between males and females, including variances in lipid metabolism, glucose homeostasis, and energy expenditure (21, 22). Furthermore, hormonal influences, such as androgens and estrogens, contribute substantially to these sex-specific metabolic differences, affecting adipose tissue distribution, insulin sensitivity, and metabolic responses to dietary challenges (22, 23).

The aim of the present study was to investigate the effects of MT_1_ removal in melatonin proficient female mice (C3H-f+/+) under a low and high-fat diet (LFD and HFD). Our study indicated that MT_1_ signaling is involved in the modulation of many of the negative effects observed under HFD.

## Materials and methods

### Animals

Melatonin-proficient (C3H-f+/+) wild-type and MT_1_ knockout (C3H-f+/+-MT1 KO) female mice were used in our study (see 15 for details). All mice were 6 weeks old at the beginning of the experiment. Mice were kept on a 12 light: 12 dark cycle, single-caged and fed with LFD (10% fat; D12450H; Research diet) or HFD (45% fat; D12451; Research diets) for 14 weeks. Once every week, mice were fasted for 6 hours, and weight, food intake and basal glucose levels were measured for all the mice until 14 weeks. All experimental procedures were performed under the NIH Guide on Care and Use of Laboratory Animals and were approved by the Morehouse School of Medicine Animal Care and Use Committee.

Sample size was determined based on the primary metabolic endpoint, with the goal of achieving statistical power >0.8 at p = 0.05. However, sample sizes differed among assays because longitudinal measures were collected from the full available cohort, whereas terminal biochemical, histological, hormonal, and RNA-seq assays depended on sample availability because a small number of mice were lost before terminal collection during the 14-week experiment. Animals were not excluded based on outcome values. Exact n values for each assay are provided in the figure legends.

Mice were singly housed during the dietary intervention to allow accurate measurement of individual weekly food intake. Group-specific n values for each assay are provided in **Table 1**. and in the corresponding figure legends.

**Table 1:** Group-specific sample sizes for each experimental endpoint.

Assay/Endpoint	WT-LFD	WT-HFD	MT1 KO-LFD	MT1 KO-HFD
Body weight	9	7	7	11
Food intake	9	7	7	11
Caloric intake	9	7	7	11
GTT	9	7	6	8
Serum NEFA	9	7	6	8
Serum cholesterol	9	7	6	8
Serum phospholipids	9	7	6	8
Serum triglycerides	9	7	6	8
ALT	4	4	4	4
Liver triglycerides/mg tissue	9	7	6	8
MASLD score	4	4	4	4
Serum estradiol	4	4	4	4
RNA-seq	3	3	3	3
Oil Red O staining	4	4	4	4

Values indicate the number of animals or biological samples analyzed in each genotype and diet group. Body weight, food intake, and caloric intake were measured during the 14-week dietary intervention, with weight gain calculated from baseline to week 14. Other endpoints were measured using the available assay-specific samples. No animals or samples were excluded based on outcome values.

### Glucose tolerance test (GTT)

To determine glucose tolerance, mice were placed in clean cages (without food) 6 h before the experiment and were injected intraperitoneally (i.p., 0.6 ml) with glucose (1.5 mg/g of body weight; 20% glucose in 0.9% NaCl). Blood glucose levels were measured before the injection of glucose and at 20, 40, 60, 80 and 100 min after injection.

Lipid profile analysis. At the end of the experiment, mice were anaesthetized with isoflurane, and a cardiac puncture was done for blood collection. After blood collection, the mice were sacrificed, and the liver was dissected out. Blood was collected and treated with 100 μL of 0.5 mol/L of ethylenediaminetetraacetic acid. The treated blood was centrifuged at 2000 g for 15 minutes at 4 °C, and the plasma (supernatant) was frozen and stored at −80 °C. Both serum and liver were sent to the Mouse Metabolic Phenotyping Center at the University of Cincinnati (Cincinnati, OH) to measure total cholesterol, triglyceride, phospholipids and non-esterified fatty acids in serum and triglyceride accumulation in the liver via Folch’s analysis (see 20 for details).

Liver Histopathology. Histopathology was performed by Premier Laboratory (Longmont, CO). Briefly, fresh mouse liver was fixed in 10% neutral formalin, embedded in paraffin, and sectioned at a 4 μm thickness. Liver sections were stained in hematoxylin and eosin (H&E). All stained sections were photographed at ×200 magnification (Leica Biosystems, USA) and had semi-quantification by Image J (NIH, USA). They were examined to assess a nonalcoholic fatty liver disease (NAFLD) activity score (NAS) based on histological features as described in (24, 25), which is now often referred to as the MASLD (metabolic dysfunction-associated fatty liver disease) activity score under updated MASLD nomenclature (26) by a trained pathologist (Speciality Vetpath, Seattle, WA). The final MASLD score comprised of a total of three categories and was classified into steatosis, including macrovascular and microvascular steatosis, scored 0–3; lobular inflammation scored 0–3; and hepatocellular ballooning scored 0–2, according to the standardized NAFLD Activity Score system described by Kleiner et al. (27), with criteria adapted for rodent NAFLD/NASH histopathology as described previously (24, 25).

### RNA sequencing

Fresh frozen livers from WT and MT1 KO mice (n=3), fed on LFD and HFD, were sent to Azenta Life Sciences (South Plainfield, USA) for RNA-sequencing. The 12 RNA-sequencing (RNA-seq) libraries were then sequenced on an Illumina HiSeq2000 platform to produce approximately 65 million 100-nucleotide paired-end reads per sample (reads 1 and 2). The reads were mapped to the University of California – Santa Cruz (UCSC; Santa Cruz, CA, USA) mouse genome assembly and transcript annotation (mm10). Mapping was performed with Bowtie2 (version 2.1.0) using the default settings. HTSeq-count (PyCharm Community Edition 2016.3.2) was used to generate counts of reads uniquely mapped to annotated genes using the UCSC mouse assembly mm10 gtf file. Further, reads per kilobase per million (RPKM) were calculated manually and only the genes having an RPKM of ≥ 1 were considered for further analysis. Fold change was later calculated by using the ratio of RPKM values of the same gene at two genotypes or feeding regime. Finally, we used a t-test (paired-end ≤ 0.05) and a cutoff of fold change ≥ 1.5 to determine differentially expressed genes (DEGs). Per-sample RNA-seq quality-control metrics, including total read pairs, GC content, read length, mapping rate, assigned counts, detected genes, and genes with RPKM >= 1, are provided in **Supplementary Table 2**. To evaluate the significance of the identified DEGs, we used String (version 12.0) analysis tools to identify significantly (FDR < 0.05) enriched pathways. For genes commonly affected by the removal of MT1 or HFD, hierarchical clustering was done using Morpheus (Broad Institute). A heat map was generated to analyze the expression pattern of these genes. Finally, a cluster profile was plotted based on gene expression.

Venn diagram analysis of RNA-seq data was performed using criteria adapted from Fougeray et al. (28). Differentially expressed gene lists were generated to identify genes associated with genotype effect or diet effect. The genotype-effect list included genes differentially expressed between WT and MT1 KO mice, regardless of diet condition. The diet-effect list included genes differentially expressed between LFD and HFD mice, regardless of genotype. Genes meeting the differential expression threshold in each category were then used for overlap analysis. Genes present only in the genotype-effect list were considered genotype-associated, genes present only in the diet-effect list were considered diet-associated, and genes present in both lists were considered shared genotype- and diet-associated genes. This analysis was used to distinguish genes primarily associated with genotype, genes primarily associated with diet, and genes shared between the two categories, thereby identifying hepatic transcriptional programs that may be influenced by both MT1 signaling and dietary challenge.

### Estrogen analysis

Serum estradiol was measured using the Mouse Estradiol Rapid ELISA Kit (Invitrogen, Cat. No. EELR013) according to the manufacturer’s instructions. This competitive ELISA quantifies mouse estradiol (E2) in serum, with a reported analytical sensitivity of 1.88 pg/mL and assay range of 3.13–200 pg/mL. The reported intra-assay and inter-assay coefficients of variation are both <10%. Serum samples were analyzed using 50 µL serum per well, and absorbance was measured at 450 nm.

### Statistical analysis

Data were analyzed using two-way ANOVA with genotype and diet as factors. Main effects of genotype, diet, and the genotype x diet interaction are reported along with the respective results. When a significant interaction or main effect was detected, Tukey’s *post hoc* test was used for multiple comparisons between groups. In case of Glucose tolerance test, curves were analyzed using two-way repeated-measures ANOVA with time and experimental group as factors, followed by *post hoc* multiple comparisons where appropriate. Differences were considered statistically significant at p ≤ 0.05 and power 0.8.

## Results

### HFD causes weight gain in WT and MT1 KO and impairs glucose metabolism in MT1 KO.

**Figure 1:**
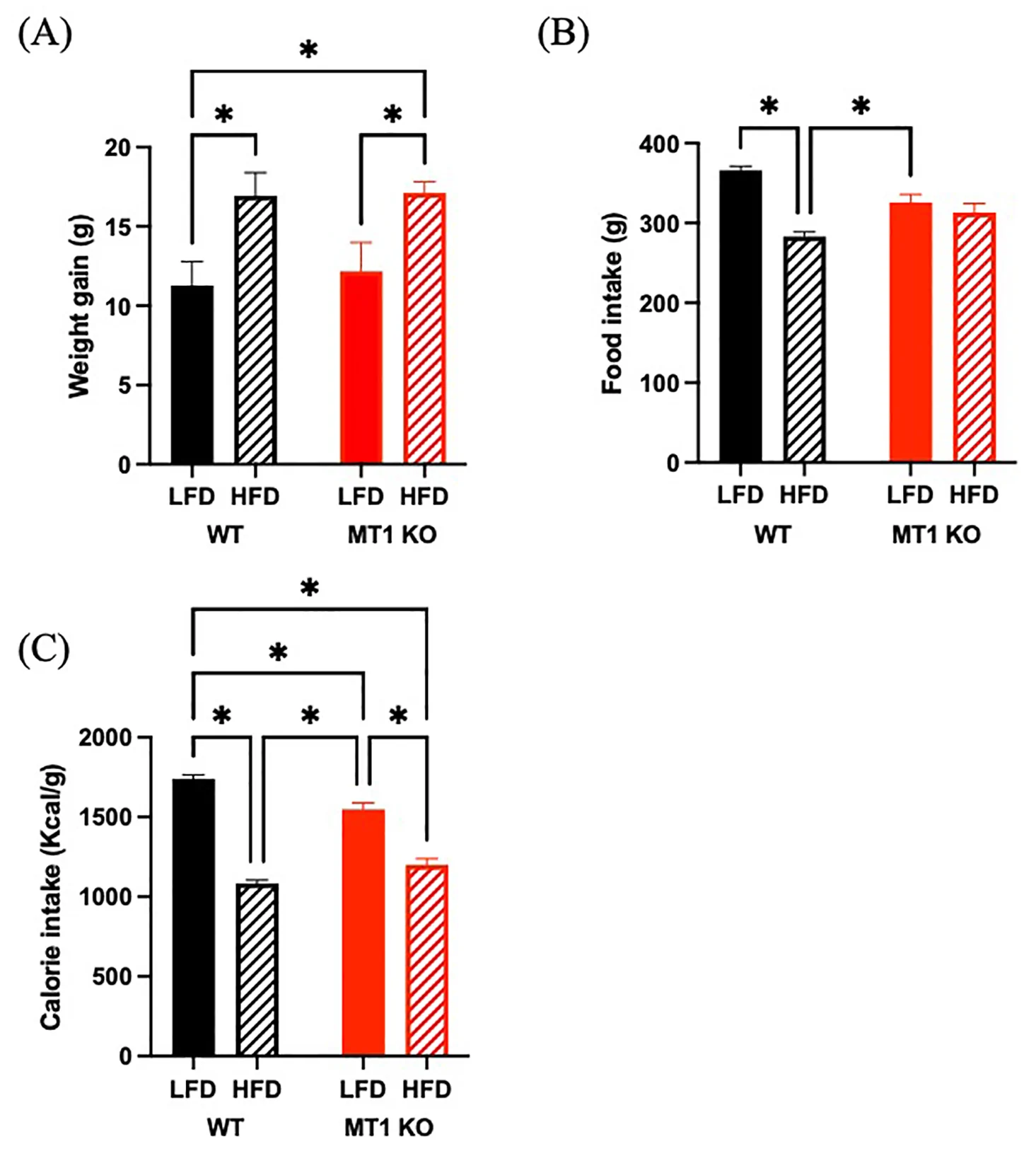
Removal of MT_1_ leads to an increase in weight gain. **(a)** Cumulative weight gain, **(b)** food intake **(c)** calorie intake. Results are expressed as mean ± SEM; **(a–c)** Two-way ANOVA Tukey’s *post hoc* test; *p < 0.05; n = 6–11.

**Figure 2:**
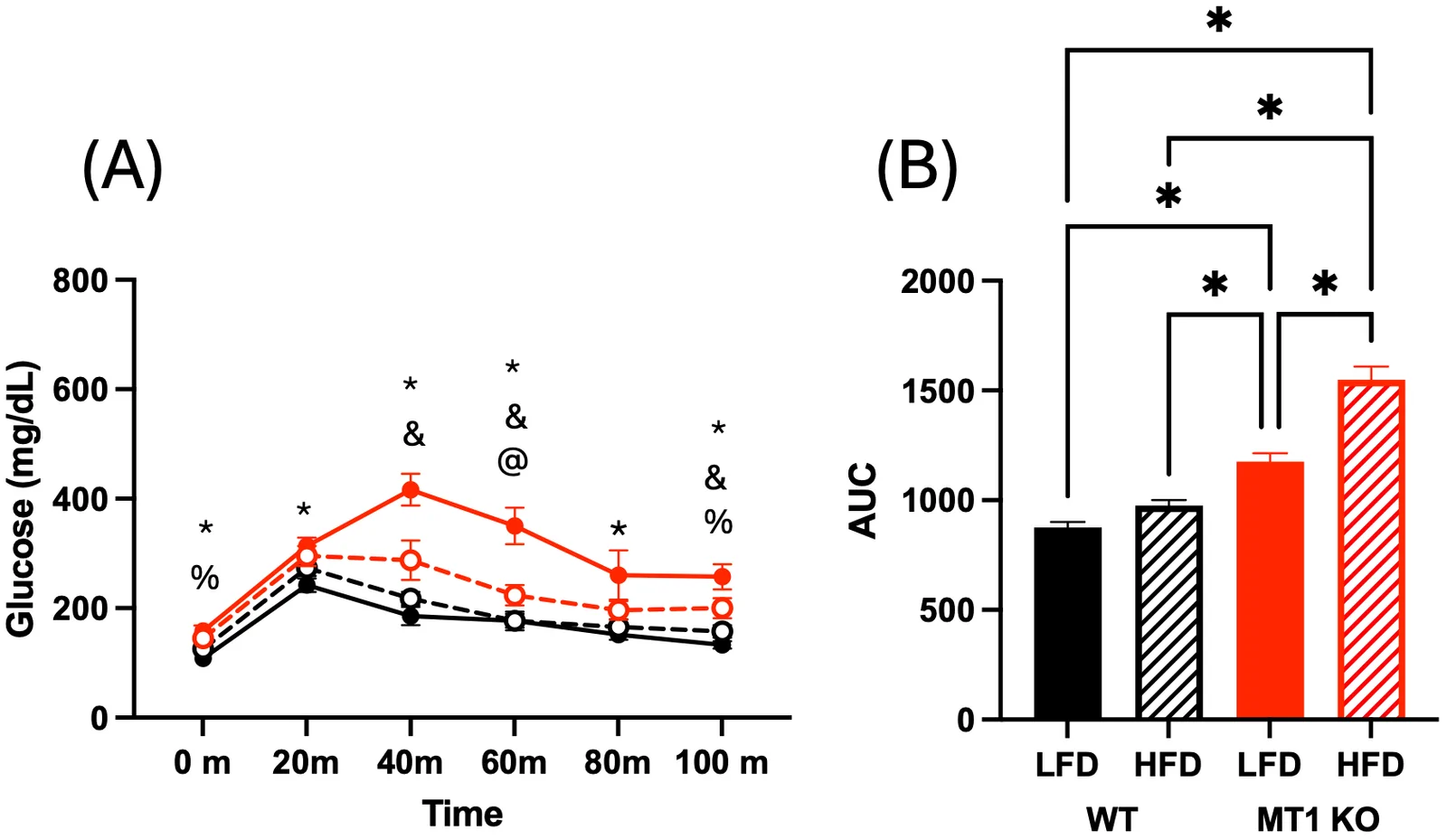
Glucose tolerance is affected by removal of MT1 signaling. Glucose tolerance was impaired in MT1 KO mice, whereas no significant change was observed in WT mice under LFD or HFD conditions **(a)**. Quantification by area under the curve (AUC) showed that the impairment was more pronounced in MT1 KO mice under HFD, although a smaller but significant increase in AUC was also observed in MT1 KO mice under LFD **(b)**. Results are expressed as mean ± SEM. GTT curves and AUC values were analyzed by two-way ANOVA followed by Tukey’s *post hoc* test. Symbols indicate significant differences between groups: *WT HFD vs. MT1 KO HFD; %WT HFD vs. MT1 KO LFD; &MT1 KO HFD vs. WT LFD; @MT1 KO HFD vs. MT1 KO LFD. p < 0.05. n = 6–9 mice per group.

To investigate the role of MT1 signaling in female mice, we monitored body weight in WT and MT1 KO mice with unrestricted access to either LFD or HFD for 14 weeks (**Figure 1A**). Two-way ANOVA showed a significant main effect of diet on body weight gain (F(1,26) = 14.24, p = 0.000840), whereas the main effect of genotype was not significant (F(1,26) = 0.1451, p = 0.706355). The diet x genotype interaction was also not significant (F(1,26) = 0.06611, p = 0.799105), indicating that HFD increased body weight similarly in WT and MT1 KO mice. Thus, HFD increased body weight independently of MT1 genotype (**Figure 1A**). Food intake by mass differed significantly between diet groups and showed a significant diet x genotype interaction. Two-way ANOVA revealed a significant main effect of diet (F(1,29) = 26.89, p = 0.000015) and a significant diet x genotype interaction (F(1,29) = 14.44, p = 0.000687), whereas the main effect of genotype was not significant (F(1,29) = 0.3039, p = 0.585648). Specifically, WT mice consumed more food under LFD than HFD, while food intake in MT1 KO mice did not differ markedly between diets (**Figure 1B**). Because the LFD and HFD differ in caloric density, we also calculated cumulative caloric intake for each group (**Figure 1C**). Two-way ANOVA showed a significant main effect of diet on caloric intake (F(1,32) = 209.6, p < 0.000001) and a significant diet x genotype interaction (F(1,32) = 19.44, p = 0.000110), whereas the main effect of genotype was not significant (F(1,32) = 1.200, p = 0.281521). Caloric intake showed the same overall trend as food intake by mass, indicating that the feeding pattern was not explained solely by differences in diet caloric density. Glucose tolerance was assessed by measuring blood glucose every 20 min for 100 min after glucose injection and analyzed by two-way repeated-measures ANOVA with time and experimental group as factors. The analysis showed significant main effects of time (F(3,25) = 18.85, p < 0.0001) and group (F(3.387,84.68) = 47.24, p < 0.0001), as well as a significant group x time interaction (F(15,125) = 4.180, p < 0.0001). These results indicate that glucose tolerance differed among experimental groups over time. HFD did not significantly impair glucose tolerance in WT mice, whereas MT1 KO mice showed reduced glucose tolerance under HFD conditions (**Figures 2A, B**). GTT responses were further quantified by calculating the area under the curve (AUC) for each mouse (**Figure 2B**). Ordinary two-way ANOVA showed significant main effects of genotype (F(1,16) = 120.2, p < 0.0001) and diet (F(1,16) = 35.48, p < 0.0001), as well as a significant diet x genotype interaction (F(1,16) = 11.91, p = 0.0033). Overall, MT1 KO mice showed higher GTT AUC values than WT mice, and HFD increased GTT AUC, with the diet-dependent increase being more pronounced in MT1 KO mice. This indicates impaired glucose tolerance in MT1 KO mice, particularly under HFD conditions.

### Removal of MT1 induces dyslipidemia and hepatic lipid accumulation under HFD.

**Figure 3:**
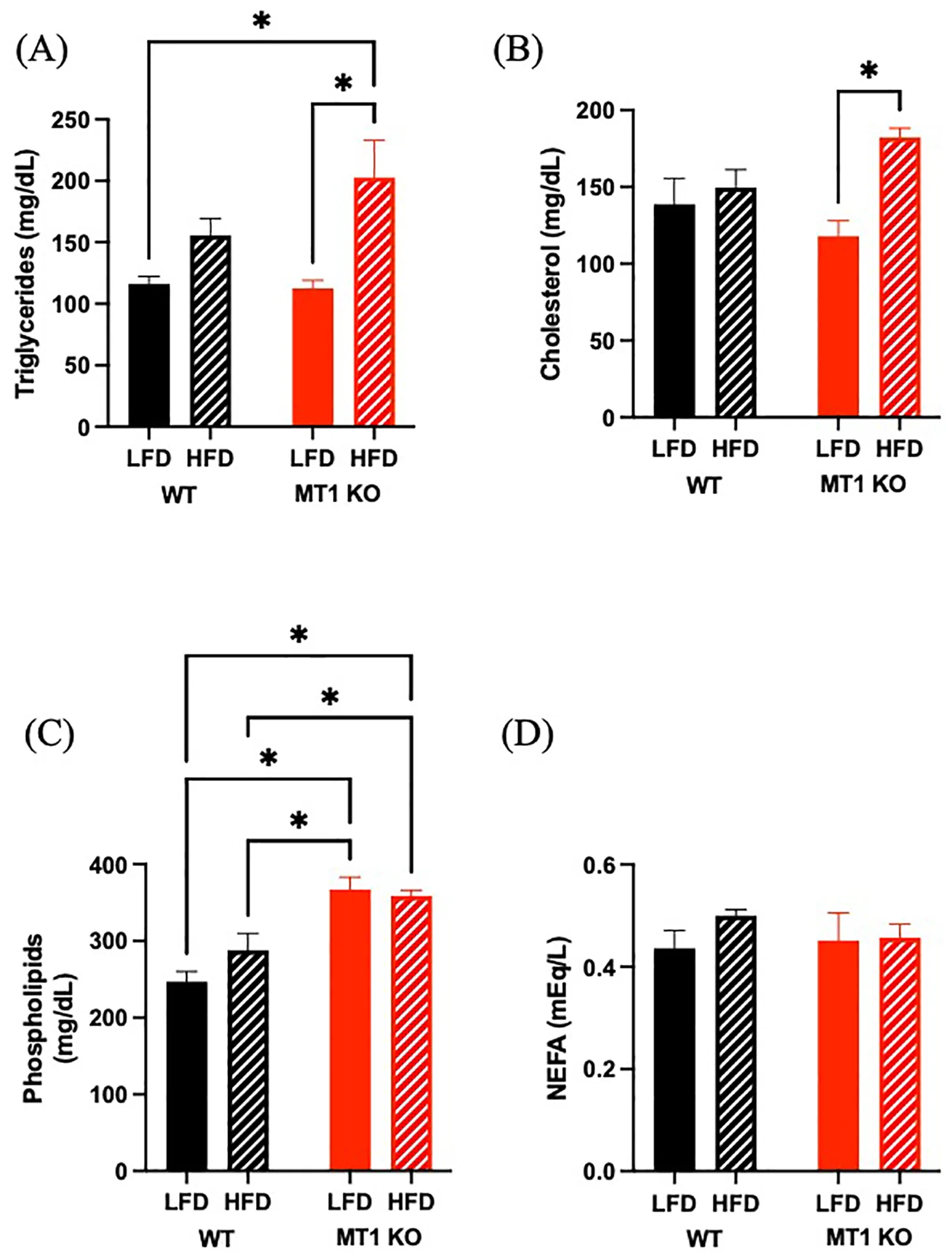
Plasma levels are affected by the removal of MT_1_. Lipid parameters were measured in the plasma of WT and MT_1_ KO mice under LFD and HFD: **(a)** triglycerides; **(b)** total cholesterol; **(c)** phospholipids, and **(d)** NEFA. Results are expressed as mean ± SEM; Two-way ANOVA Tukey’s *post hoc* test; *p < 0.05 (n = 6–9).

**Figure 4:**
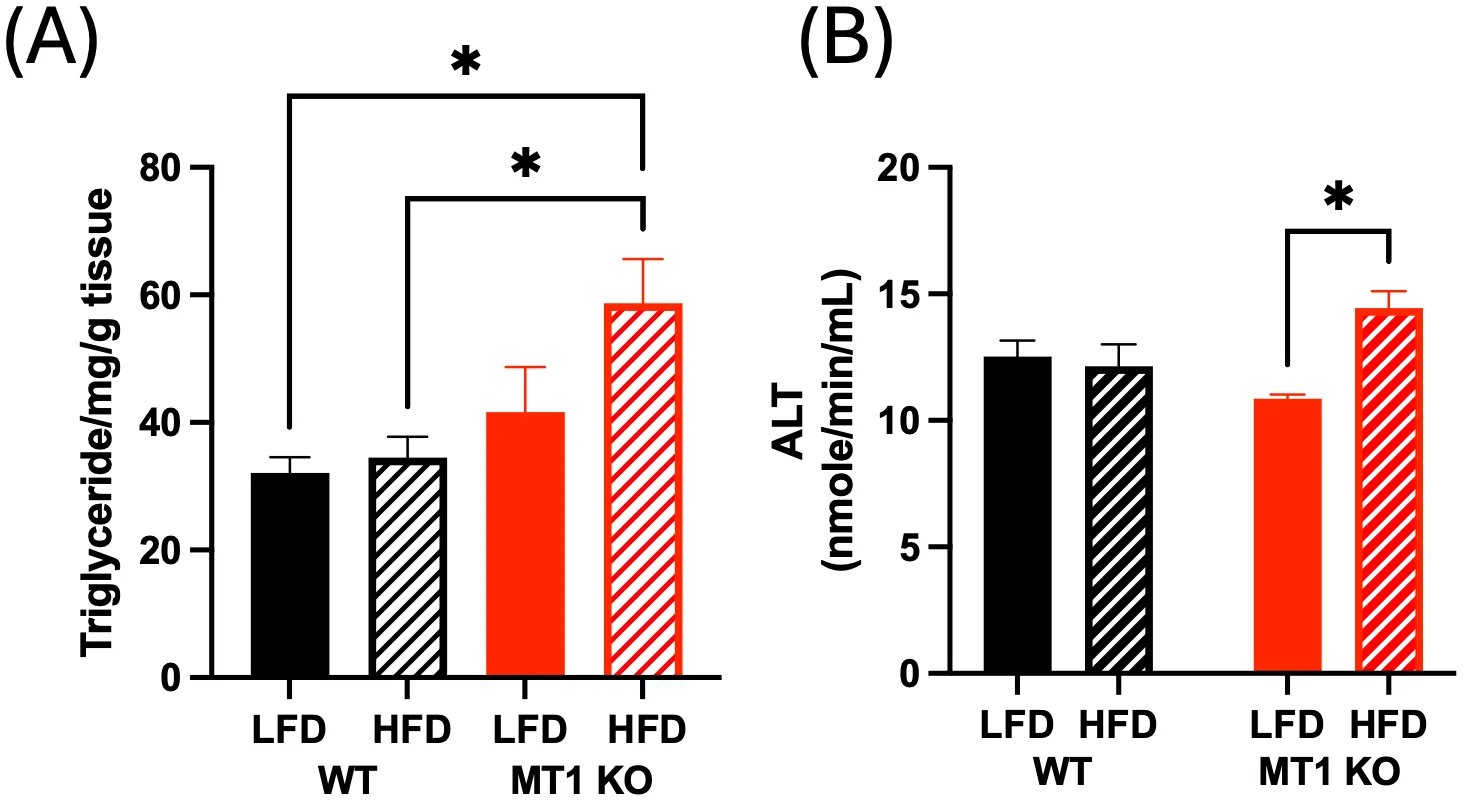
Removal of MT_1_ affects liver lipids and ALT levels. Removal of MT_1_ under HFD also leads to an increase in the accumulation of triglycerides in the liver of mice **(a)** and ALT **(b)**. Liver triglyceride deposition was assessed with Folch’s method. Data were analyzed by two-way ANOVA with genotype and diet as factors, followed by Tukey’s *post hoc* test; *p < 0.05 (n = 6–9).

**Figure 5:**
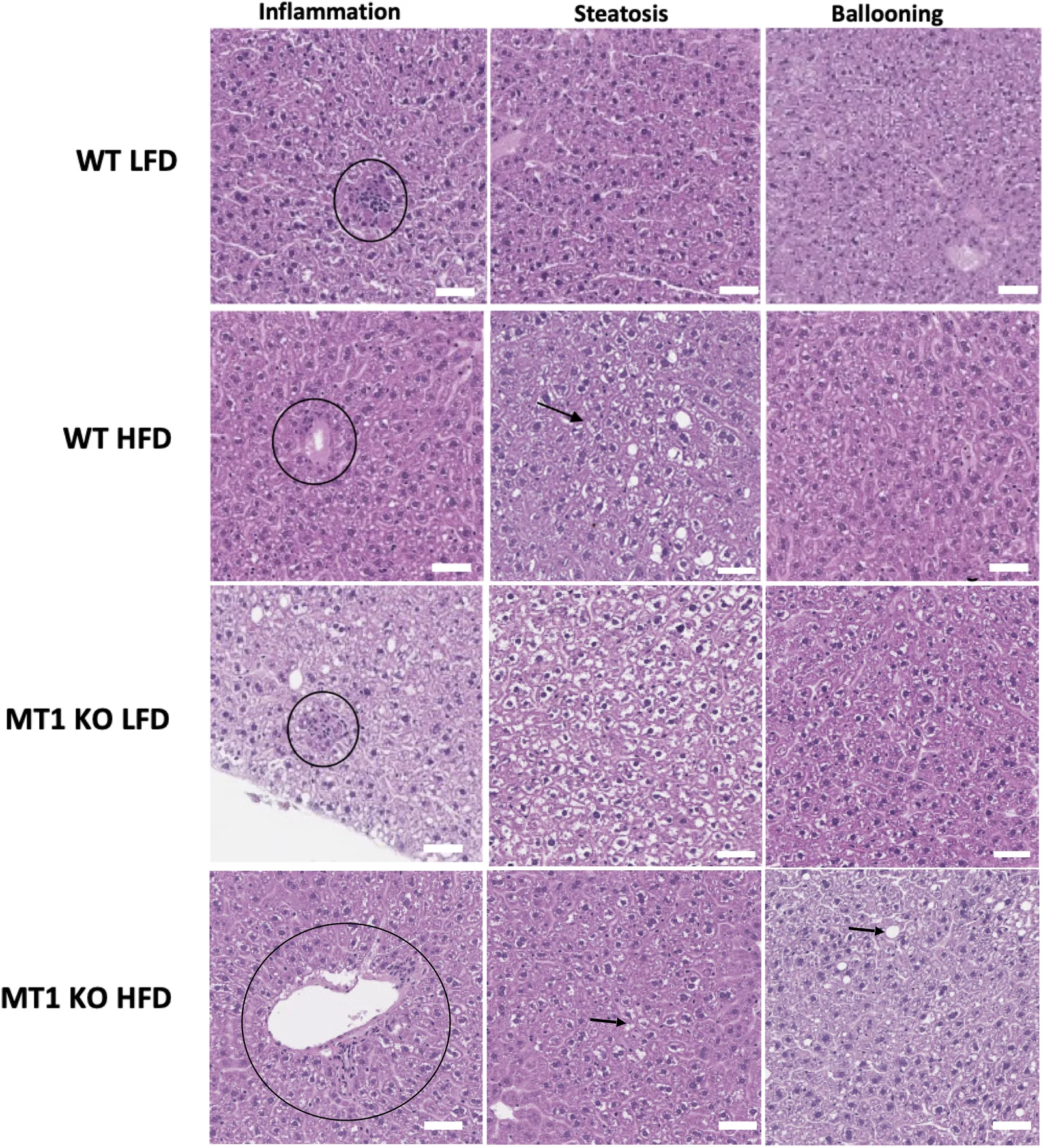
Liver histopathology in WT and in MT_1_ KO under LFD and HFD. Representative microphotographs of liver sections stained with H&E. Although in MT_1_ KOs, we observed some signs of inflammation, steatosis and ballooning as indicated by the black arrows and circles, the MASLD overall score was low. This suggests that MT_1_ deletion combined with HFD feeding does not lead to the development of MASLD, at least under our experimental conditions (see **Table 2**, n= 4, scale bar = 100 μm).

Serum lipid analysis showed that diet significantly affected all measured lipid parameters. Serum triglycerides were significantly affected by diet (F(1,16) = 4.535, p = 0.0491), whereas genotype (F(1,16) = 0.2283, p = 0.6393) and the diet x genotype interaction (F(1,16) = 0.9498, p = 0.3443) were not significant (**Figure 3A**). Serum cholesterol was significantly affected by both diet (F(1,11) = 27.65, p = 0.0003) and genotype (F(1,11) = 7.581, p = 0.0188), with higher levels observed under HFD and in MT1 KO mice, while the diet x genotype interaction was not significant (F(1,11) = 2.787, p = 0.1232) (**Figure 3B**). Similarly, serum phospholipids were significantly affected by diet (F(1,16) = 31.65, p < 0.0001) and genotype (F(1,16) = 5.936, p = 0.0269), with no significant diet x genotype interaction (F(1,16) = 0.8942, p = 0.3584) (**Figure 3C**). NEFA levels were significantly affected by diet (F(1,11) = 23.16, p = 0.0005), but not by genotype (F(1,11) = 0.1685, p = 0.6893) or the diet x genotype interaction (F(1,11) = 0.1137, p = 0.7423) (**Figure 3D**). Liver triglyceride levels were significantly affected by genotype, with higher levels in MT1 KO mice compared with WT mice (F(1,26) = 10.59, p = 0.0031). The main effect of diet did not reach significance (F(1,26) = 3.535, p = 0.0713), and the diet x genotype interaction was not significant (F(1,26) = 2.016, p = 0.1676). These results indicate that MT1 deletion was associated with increased hepatic triglyceride accumulation, with the highest levels observed in MT1 KO mice under HFD conditions (**Figure 4A**; **Supplementary Figure 1**). Serum alanine aminotransferase (ALT) levels were significantly affected by diet (F(1,12) = 6.347, p = 0.026943) and showed a significant diet x genotype interaction (F(1,12) = 9.660, p = 0.009054), whereas the main effect of genotype was not significant (F(1,12) = 0.2466, p = 0.628429). ALT levels were increased under HFD, with the diet-dependent change differing between WT and MT1 KO mice (**Figure 4B**). Histopathological analysis of liver sections obtained from mice under different feeding regimes indicated no significant difference in the level of inflammation and ballooning, while the steatosis score was slightly elevated in MT1 KO under HFD (see **Figure 5**; **Table 2**). However, the overall NAS score was low in all samples, suggesting that under our experimental conditions, removal of MT1 does not lead to MASLD in female mice.

**Table 2:** Semiquantitative scoring of liver histopathology.

Group	Inflammation	Steatosis	Ballooning
WT LFD	0.75 ± 0.25	0.0 ± 0.0	0.25 ± 0.25
MT_1_ KO LFD	0.25 ± 0.25	0.0 ± 0.0	0.0 ± 0.0
WT HFD	0.50 ± 0.28	0.25 ± 0.25	0.0 ± 0.0
MT_1_ KO HFD	0.50 ± 0.28	1.25 ± 0.47^a,b,c^	0.25 ± 0.25

Semi-quantitative histopathological scoring of liver sections. Liver inflammation, steatosis, and hepatocellular ballooning were scored in H&E-stained liver sections from WT and MT1 KO mice fed LFD or HFD. Values are expressed as mean +/- SEM; n = 4 mice per group. a, b and c represent the p < 0.05 (n = 4) in cases of MT1 KO HFD vs. WT LFD, MT_1_ KO HFD vs. MT_1_ KO LFD, and MT_1_ KO HFD vs. WT HFD.

### Analysis of the liver transcriptome

To explore the transcriptional programs that may contribute to the physiological, metabolic, biochemical, and histological changes observed in WT and MT1 KO mice under LFD and HFD conditions, we performed RNA-seq analysis of liver tissue as an exploratory approach. 11,536 genes were expressed in the liver and among these genes, 5,866 were differentially expressed among the different genotypes and/or feeding regimes. A large set of genes (2,410) were differentially expressed between WT and MT_1_ KO mouse liver under LFD or HFD. Conversely, 2,453 genes were sensitive to diet (LFD versus HFD) with little influence from MT_1_ deletion. Finally, 1,003 genes were sensitive to both MT_1_ deletion and HFD.

**Figure 6:**
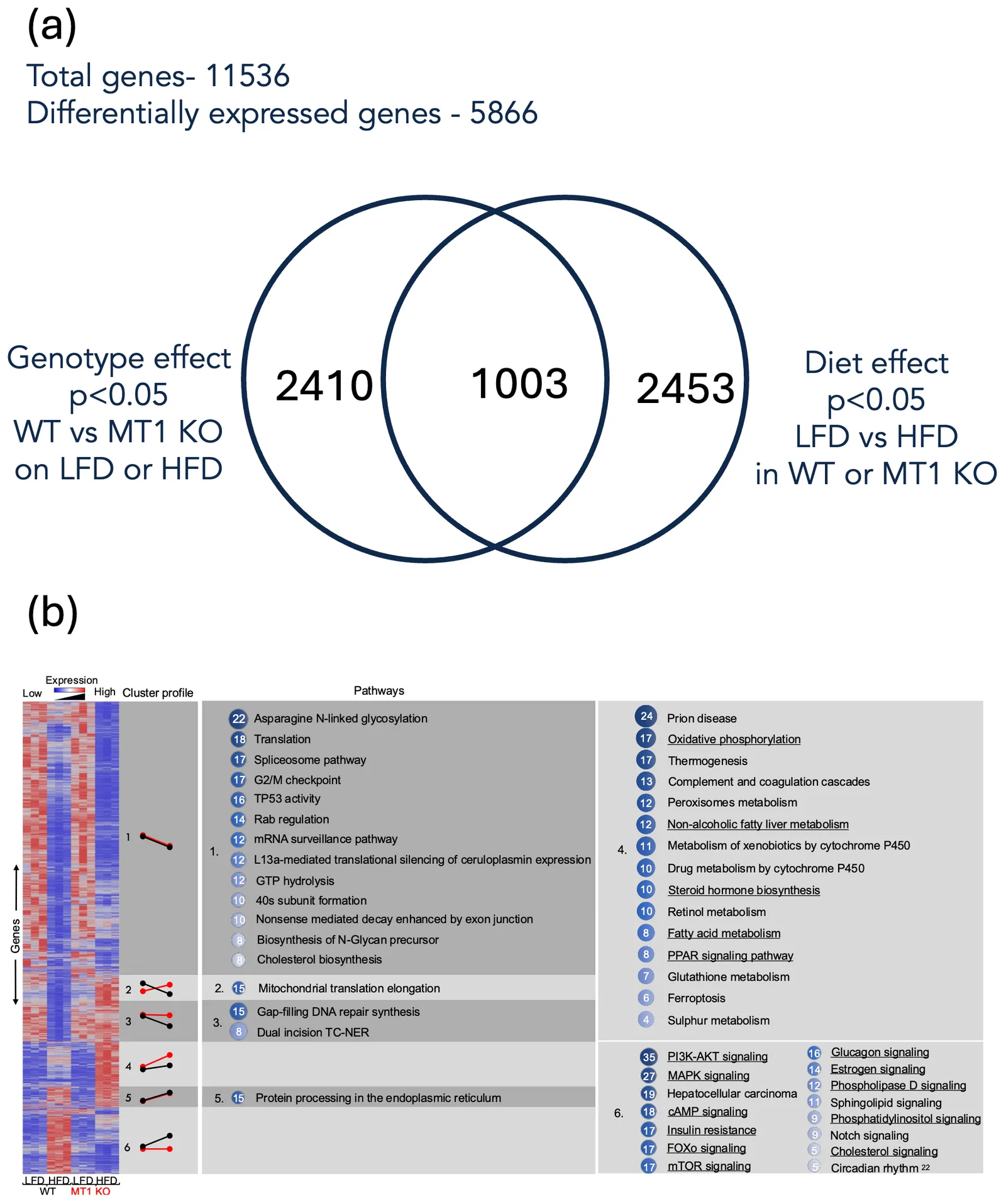
Venn diagram and hierarchical clustering analysis of genes affected by MT1 deletion and HFD. **(a)** Venn diagram showing differentially expressed genes associated with genotype effect, diet effect, or both. The genotype-effect category includes genes differentially expressed between WT and MT1 KO mice under LFD or HFD, whereas the diet-effect category includes genes differentially expressed between LFD and HFD within WT or MT1 KO mice. **(b)** Heat map showing hierarchical clustering of differentially expressed hepatic genes affected by genotype, diet, or both. Schematic cluster profiles are shown next to the heat map, where black lines represent WT mice and red lines represent MT1 KO mice. The dots at the start and end of each line represent the LFD and HFD groups, respectively (upward, downward, and horizontal lines indicate increased, decreased, or unchanged expression with HFD, respectively). Pathway analysis was performed for the genes within each cluster. Gene-numbers; The circle color gradient represents pathway enrichment levels. n = 3 mice per group.

To further investigate these results, we performed hierarchical clustering on these 5,866 differentially expressed genes and identified 6 gene expression clusters (**Figure 6**).

Cluster 1 comprises genes that are repressed in response to a HFD in WT and MT1 KO livers. These genes are associated with pathways involved in glycosylation, translation, and cell-cycle checkpoints. Cluster 2 comprises genes that in WT and under HFD exposure led to downregulation of these genes, whereas in MT_1_ KO, the same dietary condition induced an upregulation. Interestingly, many of these genes localize within the mitochondrial complex and are involved in mitochondrial translation elongation and termination. Cluster 3 also contains genes that are not affected by MT_1_ deletion under LFD, whereas in WT under HFD they are down regulated. The action of these genes was associated with pathways like gap-filling DNA repair synthesis and ligation. Cluster 4 comprises genes that upregulated in response to HFD in both genotypes. However, the upregulation observed in MT_1_ KO mice is higher than in WT. Genes in this cluster are associated with prion disease, oxidative phosphorylation, thermogenesis, MASLD, steroid hormone biosynthesis, fatty acid metabolism, PPAR signaling, and ferroptosis. Cluster 5 includes genes that are not differentially expressed between the two genotypes under LFD or under HFD, and these genes are involved in pathways such as protein processing in the endoplasmic reticulum. Finally, Cluster 6 contains genes that are upregulated in WT under HFD, but not in MT_1_ KO. The pathways affected in this cluster are PI3K-AKT signaling, MAPK signaling, cAMP signaling, insulin resistance, FoxO signaling, mTOR signaling, glucagon signaling, estrogen signaling, phospholipase D signaling, phosphatidylinositol signaling and cholesterol signaling.

### Estrogen levels are upregulated in WT, but not in MT1 KO under HFD.

**Figure 7:**
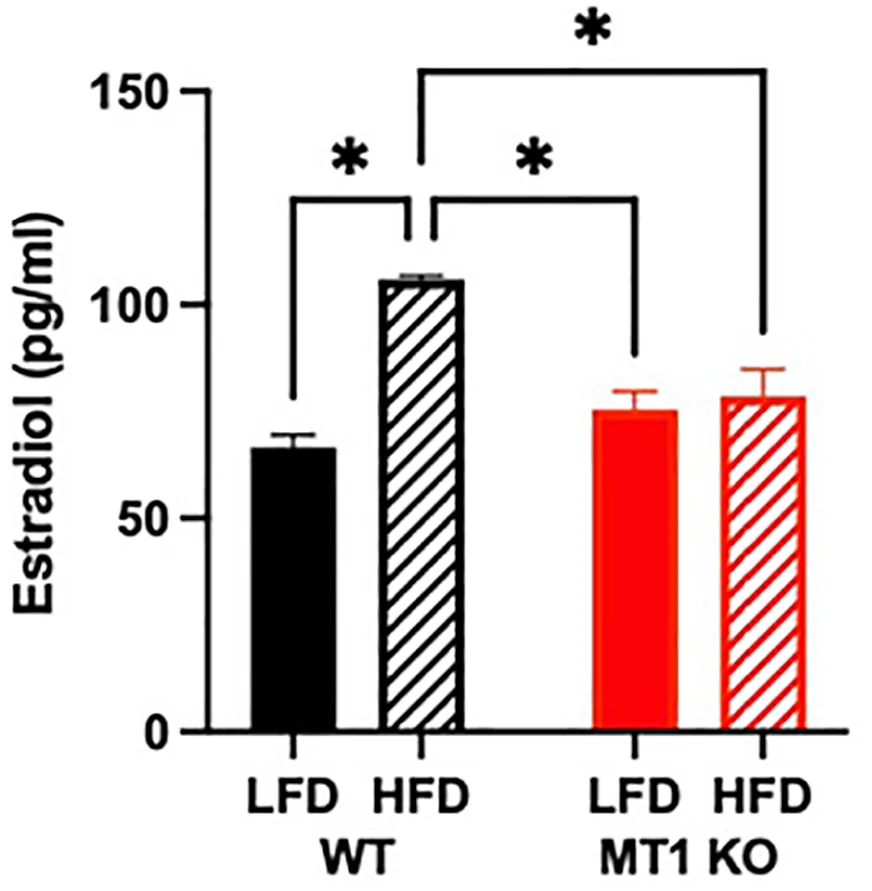
Estradiol (E2) levels are affected by removal of MT1 under HFD. Estradiol levels were elevated in WT mice under HFD, whereas estradiol levels in MT_1_ KO do not change under HFD. Results are expressed as mean ± SEM; two-way ANOVA followed by Tukey’s *post hoc* test; *p < 0.05 (n = 4).

Circulating 17beta-estradiol (E2) levels were significantly affected by diet and genotype and showed a significant diet x genotype interaction. Two-way ANOVA showed a significant main effect of diet (F(1,12) = 26.21, p = 0.000254), a significant main effect of genotype (F(1,12) = 4.877, p = 0.047409), and a significant diet x genotype interaction (F(1,12) = 18.93, p = 0.000943). Overall, E2 levels were higher under HFD than LFD and were higher in WT than MT1 KO mice. The significant interaction indicates that the diet-dependent change in circulating E2 differed between genotypes, with WT-HFD mice showing higher E2 levels than MT1 KO-HFD mice (**Figure 7**).

## Discussion

The present study aimed to elucidate the impact of MT_1_ removal on melatonin-proficient female mice under LFD and HFD. Our findings demonstrate that MT_1_ deletion alone does not cause notable changes in weight, lipid profile or hepatic lipid accumulation, whereas the combination of MT_1_ deficiency and HFD exposure results in significant changes in glucose tolerance, serum lipid profiles, and lipid accumulation in the liver. In addition, removal of MT_1_ signaling also induced significant changes in the liver transcriptome that was exacerbated by exposure to HFD. Finally, our data indicated that removal of MT_1_ signaling affects estrogen signaling and serum levels.

Our observation that HFD induces weight gain in both WT and MT1 KO mice, independent of MT1 deletion (**Figure 1A**), aligns with previous studies highlighting the strong obesogenic effects of HFD (29, 30). Interestingly, while food intake remained consistent across groups, the marginally lower intake in WT mice on HFD suggests a possible adaptive response to diet composition, which is an effect that appears diminished in MT1 KO mice (**Figure 1B**). This indicates that MT1 signaling may play a role in dietary energy sensing and weight regulation, potentially modulating metabolic rate or energy expenditure (17, 31). Because diets differed in caloric density, caloric intake was calculated in addition to food intake by mass. The caloric intake analysis showed the same overall trend as the gram-based food intake data, supporting the interpretation that group differences in feeding behavior were not solely due to diet energy density.

Previous studies have shown that in male mice, removal of MT_1_ signaling induces significant changes in glucose tolerance under “normal” feeding conditions (15, 16) and such an effect is exacerbated in mice subjected to HFD (32). The current study showed that in female mice, the removal of MT_1_ under LFD has only a modest, but significant effect, on glucose metabolism. This effect became much more pronounced once the mice were exposed to HFD (**Figure 2**). Hence, our data support the idea that MT_1_ signaling may protect against HFD-induced glucose dysregulation.

An important observation in this study is that WT female mice did not develop marked glucose intolerance after HFD exposure, despite increased body weight and metabolic challenge. This relative preservation of glucose tolerance is consistent with the known resistance of female mice to diet-induced metabolic dysfunction compared with males and may reflect protective effects of ovarian hormone signaling and other sex-dependent metabolic adaptations. In contrast, MT1 KO females showed impaired glucose tolerance under HFD, suggesting that loss of MT1 signaling may reduce this metabolic resilience and increase susceptibility to HFD-induced glucose dysregulation. However, because estrous cycle stage was not monitored, the contribution of estrogen signaling to this phenotype remains exploratory and requires further study.

Furthermore, the development of dyslipidemia, characterized by elevated serum triglyceride and cholesterol levels, was notable only in MT_1_ KO mice under HFD (**Figure 3**). This finding aligns with studies linking melatonin signaling to lipid metabolism and an ability to reduce lipogenesis and promote lipid oxidation (18, 20, 33).

Consistent with the biochemical findings of dyslipidemia, our data further demonstrated enhanced hepatic lipid accumulation in HFD-fed MT_1_ KO mice liver, along with a slight elevation of ALT (**Figure 5**). However, the histological analysis of liver sections did not show any clear pathology, as indicated by the low MASLD score (**Table 2**) in both genotypes and feeding regime. Thus, it appears that although a lack of MT_1_ signaling and HFD leads to lipid accumulation and increased levels of ALT in the liver, these increases do not trigger MASLD. It is possible that this lack of effects may be due to the relative short duration of our experiment (e.g., 14 weeks) and a longer exposure to an HFD regime may lead to MASLD in MT_1_ KO mice.

Our analysis of the liver transcriptome identified multiple gene clusters and several candidate pathways modulated by diet and/or MT_1_ receptor status. Cluster 1 and Cluster 5 show a large group of genes that are not differently expressed in WT and MT_1_ under the two feeding regimes. These genes are involved in glycosylation, translation, cell cycle checkpoints, and protein processing in the endoplasmic reticulum. Since the expression of these genes do not change according to the genotype or feeding regime, we believe that these genes are not major players in the modulation of the effects observed in our studies.

Cluster 2 contains a small group of genes that have a differential level of expression between WT (high) and MT_1_ KO (low) under LFD and once exposed to HFD they show downregulation in WT and upregulation in MT1 KO. These genes are involved in mitochondrial functions and changes in their expression pattern may lead to compromised mitochondrial function, thus increasing oxidative stress and lipid accumulation in the liver (20, 34). The DEGs may play a role in the dyslipidemia observed in MT_1_ KO under HFD. Cluster 3 represents a group of genes that do not show any differential expression between the two genotypes under LFD, but under HFD they are significantly downregulated in WT. For example, the transcription-coupled nucleotide excision repair (TC-NER) pathway is essential for maintaining genomic stability, mitochondrial function, and cellular stress responses. HFD promotes oxidative stress and lipid accumulation in hepatocytes, leading to DNA damage that TC-NER repairs, especially in actively transcribed genes (35, 36). Hence, the upregulation of these in WT under HFD may reduce oxidative stress, preserve mitochondrial integrity, and thus prevent DNA damage buildup in hepatocytes (37). Melatonin via MT_1_ also modulates autophagy and mitochondrial dynamics, both of which intersect with TC-NER function, potentially strengthening liver resilience against HFD-induced stress. Without the protective support of MT_1_ signaling, the TC-NER pathway becomes overburdened, resulting in unresolved DNA damage, heightened inflammation, and lipid dysregulation.

Cluster 4 contains a large group of genes involved in key hepatic processes, including oxidative phosphorylation, fatty acid metabolism, PPAR signaling, ferroptosis-related pathways, and pathways associated with MASLD progression. In WT mice, HFD showed a blunted upregulation of this transcriptional response, whereas MT1 KO mice with HFD stronger increase in Cluster 4 gene expression. This indicates that MT1 signaling is required for the normal hepatic transcriptional response to HFD. The higher upregulation of these metabolic and stress-response pathways in MT1 KO mice provides a probable mechanistic explanation for the greater hepatic lipid accumulation and metabolic impairment observed in MT1 KO mice under HFD. Thus, Cluster 4 supports the conclusion that MT1 signaling protects against HFD-induced liver dysfunction by maintaining appropriate regulation of hepatic lipid metabolism, mitochondrial function, and stress-response pathways.

Cluster 6 also includes a larger group of DEGs that belong to many different signaling pathways which show a different level of expression between the two genotypes under HFD. In WT mice under HFD, these pathways show strong upregulation, whereas no change is present in MT_1_ KO. This suggests that upregulation of these pathways plays a protective role in WT mice when exposed to HFD. Among these groups, we found signaling pathways that are known to be involved in MT_1_ signaling (e.g., PI3K-AKT, MAPK, cAMP, FoxO, mTOR and Phosphatidylinositol) and pathways that can be linked to the results obtained in our studies (e.g., Insulin resistance glucagon, and cholesterol signaling). Finally, estrogen signaling was identified as a pathway greatly affected by lack of MT_1_ signaling under HFD. This is of particular interest since estrogen is known to exert protective effects on hepatic lipid metabolism and insulin sensitivity (38). Moreover, previous studies have also shown that MT_1_ is intricately linked to estrogen signaling, with interactions that influence hormone production, receptor activity, and downstream signaling pathways (39, 40). Although direct evidence for hepatic MT1-estrogen crosstalk in HFD-induced liver disease remains limited, prior studies support biological convergence between melatonin/MT1 signaling, estrogen-responsive pathways, and hepatic lipid metabolism. Melatonin/MT1 signaling has been implicated in hepatic mitochondrial regulation, melatonin improves experimental NAFLD and estrogen-deficiency-associated hepatic steatosis, and hepatic ERα is known to regulate glucose and lipid metabolism (41–45). Therefore, the estrogen-related pathway changes observed in the present exploratory RNA-seq analysis may represent a candidate mechanism linking MT1 signaling to the female hepatic response to HFD. Consistent with these observations, our transcriptomic and hormonal data, indicate that under HFD, the lack of MT_1_ signaling can produce a reduction in estrogen signaling, and in estrogen levels in the serum of MT_1_ KO which can upon further investigation may help explain an associated mechanism by which removal of MT_1_ leads to a disruption in glucose metabolism and lipid processing, as observed in our investigation.

A limitation of this study is that estrous cycle stage was not assessed at blood collection or sacrifice; therefore, the observed changes in estrogen-related signaling and circulating estradiol should be interpreted as supportive evidence of an association between MT1 signaling and the metabolic response to HFD, rather than as direct evidence of MT1-dependent regulation of estrogen production. Future studies incorporating estrous cycle staging and direct validation of estrogen signaling pathways will be required to determine causality. To explore whether the liver could contribute to the observed circulating estradiol differences, we examined hepatic Cyp19a1/aromatase expression in the RNA-seq dataset. Cyp19a1 was not detected in liver samples from any group, suggesting that the hepatic estrogen-related transcriptional signal is unlikely to reflect local hepatic estradiol biosynthesis. Instead, these data are more consistent with altered hepatic responsiveness to circulating estradiol. In support of this interpretation, hepatic Esr1 was detected in the RNA-seq dataset and showed a pattern broadly consistent with the circulating estradiol results (**Supplementary Table 1**). An additional limitation is that potential compensatory MT2 signaling was not directly assessed. We examined hepatic Mtnr1b expression in the RNA-seq dataset and found no evidence of significant MT2 transcriptional upregulation (log2FC = 0.2585, p = 0.410). However, RNA-seq does not assess MT2 protein abundance, receptor activity, or potential compensation in other metabolically relevant tissues. Therefore, compensatory MT2 signaling cannot be fully excluded. Together, these findings suggest that WT female mice mount an estrogen-associated adaptive response to HFD, reflected by increased circulating estradiol and increased hepatic Esr1 expression. In contrast, MT1 KO females under HFD show a blunted estrogen-associated response, with lower circulating estradiol and lower hepatic Esr1 expression compared with WT-HFD mice. This impaired estrogen-associated response may contribute to the greater metabolic vulnerability of MT1 KO females under HFD conditions.

In conclusion, MT1 signaling contributes to the metabolic and hepatic response to HFD in female mice. Loss of MT1 was associated with impaired glucose tolerance, dyslipidemia, hepatic lipid accumulation, and altered hepatic transcriptional pathways, including estrogen-related signaling. Together with the transcriptomic analysis, these findings suggest a potential association between MT1 signaling and estrogen-associated metabolic regulation in female mice. However, because this study was limited to females, our data do not establish whether estradiol mediates melatonin effects through MT1 receptors or whether these responses are sex-specific. Future studies comparing male and female mice, incorporating estrous cycle staging and direct molecular validation, are required to establish causality.

## Data Availability

The RNA-seq data are available in GEO under accession GSE344927 at the direct link above. All other supporting data are included in the article and **Supplementary Material**.
