# Supplementary material for: Melatonin receptor 1 signaling modulates the metabolic response to high-fat diet in female melatonin-proficient mice
Source: Front Endocrinol (Lausanne). 2026 Sep 8;17:1884802. doi: 10.3389/fendo.2026.1884802 (PMC13593444; doi:10.3389/fendo.2026.1884802)
Supplement: Supplementary file 1 [file Table1.docx]

Table 1S.

| **Gene** | **Annotation** | **WT-CD mean RPKM +/- SEM** | **WT-HFD mean RPKM +/- SEM** | **MT1-CD mean RPKM +/- SEM** | **MT1-HFD mean RPKM +/- SEM** |
| --- | --- | --- | --- | --- | --- |
| Cyp19a1 | Aromatase / estrogen biosynthesis enzyme | 0.0000 +/- 0.0000 | 0.0000 +/- 0.0000 | 0.0000 +/- 0.0000 | 0.0000 +/- 0.0000 |
| Esr1 | Estrogen receptor alpha | 5.5690 +/- 1.0688 | 10.0082 +/- 0.8845 | 3.9352 +/- 0.3896 | 8.5359 +/- 0.8546 |
